# Supplementary material for: Multi‐dataset identification of innovative feature genes and molecular mechanisms in keratoconus
Source: J Cell Mol Med. 2024 Sep 19;28(18):e70079. doi: 10.1111/jcmm.70079 (PMC11412914; doi:10.1111/jcmm.70079)
Supplement: Supplementary file 2 — Material S2. [file JCMM-28-e70079-s002.zip › 7.GSEA analysis/4.MYRF/KEGG_STARCH_AND_SUCROSE_METABOLISM.html]

Details for gene set KEGG\_STARCH\_AND\_SUCROSE\_METABOLISM[GSEA]

|  || Dataset | MYRF.group.cls#h\_versus\_l |
| Phenotype | group.cls#h\_versus\_l |
| Upregulated in class | h |
| GeneSet | KEGG\_STARCH\_AND\_SUCROSE\_METABOLISM |
| Enrichment Score (ES) | 0.24298377 |
| Normalized Enrichment Score (NES) | 0.73464614 |
| Nominal p-value | 0.8102041 |
| FDR q-value | 1.0 |
| FWER p-Value | 1.0 |
Table: GSEA Results Summary

  

Fig 1: Enrichment plot: KEGG\_STARCH\_AND\_SUCROSE\_METABOLISM      
 Profile of the Running ES Score & Positions of GeneSet Members on the Rank Ordered List

  

| SYMBOL | TITLE | RANK IN GENE LIST | RANK METRIC SCORE | RUNNING ES | CORE ENRICHMENT || 1 | UGT1A8 | na | 369 | 0.620 | 0.1010 | Yes |
| 2 | HK3 | na | 1529 | 0.399 | 0.1394 | Yes |
| 3 | UGT1A6 | na | 2556 | 0.320 | 0.1675 | Yes |
| 4 | UGT2B4 | na | 3822 | 0.260 | 0.1779 | Yes |
| 5 | GBA3 | na | 4950 | 0.224 | 0.1857 | Yes |
| 6 | ENPP3 | na | 5873 | 0.196 | 0.1945 | Yes |
| 7 | PYGM | na | 5914 | 0.195 | 0.2285 | Yes |
| 8 | UGT2B11 | na | 6521 | 0.178 | 0.2430 | Yes |
| 9 | UGT2B28 | na | 7657 | 0.147 | 0.2368 | No |
| 10 | GANC | na | 8442 | 0.128 | 0.2373 | No |
| 11 | GYS1 | na | 10145 | 0.093 | 0.2050 | No |
| 12 | GYS2 | na | 10220 | 0.092 | 0.2194 | No |
| 13 | PYGB | na | 12098 | 0.062 | 0.1765 | No |
| 14 | UGT2B10 | na | 12166 | 0.061 | 0.1856 | No |
| 15 | GAA | na | 12975 | 0.051 | 0.1714 | No |
| 16 | PGM2 | na | 15141 | 0.026 | 0.1137 | No |
| 17 | UGT2A3 | na | 16075 | 0.017 | 0.0899 | No |
| 18 | AGL | na | 16432 | 0.013 | 0.0821 | No |
| 19 | UGT2B7 | na | 16473 | 0.013 | 0.0833 | No |
| 20 | PYGL | na | 17778 | 0.002 | 0.0460 | No |
| 21 | UXS1 | na | 18310 | -0.004 | 0.0313 | No |
| 22 | GUSB | na | 18367 | -0.004 | 0.0304 | No |
| 23 | UGP2 | na | 19571 | -0.017 | -0.0012 | No |
| 24 | UGDH | na | 19767 | -0.019 | -0.0034 | No |
| 25 | GBE1 | na | 20614 | -0.029 | -0.0226 | No |
| 26 | HK1 | na | 22330 | -0.050 | -0.0631 | No |
| 27 | TREH | na | 22512 | -0.052 | -0.0588 | No |
| 28 | AMY1C | na | 23232 | -0.063 | -0.0682 | No |
| 29 | G6PC2 | na | 24433 | -0.083 | -0.0879 | No |
| 30 | GPI | na | 24601 | -0.086 | -0.0772 | No |
| 31 | PGM1 | na | 25162 | -0.096 | -0.0760 | No |
| 32 | HK2 | na | 25911 | -0.111 | -0.0776 | No |
| 33 | UGT2B15 | na | 26025 | -0.113 | -0.0605 | No |
| 34 | SI | na | 28200 | -0.166 | -0.0932 | No |
| 35 | UGT2A1 | na | 29490 | -0.205 | -0.0934 | No |
| 36 | PGM2L1 | na | 30646 | -0.248 | -0.0820 | No |
| 37 | ENPP1 | na | 31660 | -0.299 | -0.0574 | No |
| 38 | GCK | na | 32098 | -0.323 | -0.0118 | No |
| 39 | MGAM | na | 33801 | -0.486 | 0.0267 | No |
Table: GSEA details [plain text format]

  

Fig 2: KEGG\_STARCH\_AND\_SUCROSE\_METABOLISM      
 Blue-Pink O' Gram in the Space of the Analyzed GeneSet

  

Fig 3: KEGG\_STARCH\_AND\_SUCROSE\_METABOLISM: Random ES distribution      
 Gene set null distribution of ES for **KEGG\_STARCH\_AND\_SUCROSE\_METABOLISM**
